# Science-based health innovation in Ghana: health entrepreneurs point the way to a new development path

**DOI:** 10.1186/1472-698X-10-S1-S2

**Published:** 2010-12-13

**Authors:** Sara Al-Bader, Abdallah S Daar, Peter A Singer

**Affiliations:** 1 McLaughlin-Rotman Centre for Global Health, at the University Health Network and University of Toronto, MaRS Centre, South Tower, Suite 406, 101 College Street, Toronto, Ontario, M5G 1L7, Canada

## Abstract

**Background:**

Science, technology and innovation have long played a role in Ghana’s vision for development, including in improving its health outcomes.  However, so far little research has been conducted on Ghana’s capacity for health innovation to address local diseases. This research aims to fill that gap, mapping out the key actors involved, highlighting examples of indigenous innovation, setting out the challenges ahead and outlining recommendations for strengthening Ghana’s health innovation system.

**Methods:**

Case study research methodology was used. Data were collected through reviews of academic literature and policy documents and through open-ended, face-to-face interviews with 48 people from across the science-based health innovation system. Data was collected over three visits to Ghana from February 2007 to August 2008, and stakeholders engaged subsequently.

**Results:**

Ghana has strengths which could underpin science-based health innovation in the future, including health and biosciences research institutions with strong foreign linkages and donor support; a relatively strong regulatory system which is building capacity in other West African countries; the beginnings of new funding forms such as venture capital; and the return of professionals from the diaspora, bringing expertise and contacts. Some health products and services are already being developed in Ghana by individual entrepreneurs, which are innovative in the sense of being new to the country and, in some cases, the continent. They include essential medicines, raw pharmaceutical materials, new formulations for pediatric use and plant medicines at various stages of development.

**Conclusions:**

While Ghana has many institutions concerned with health research and its commercialization, their ability to work together to address clear health goals is low. If Ghana is to capitalize on its assets, including political and macroeconomic stability which underpin investment in health enterprises, it needs to improve the health innovation environment through increasing support for its small firms; coordinating policies; and beginning a dialogue with donors on how health research can create locally-owned knowledge and be more demand-driven. Mobilizing stakeholders around health product development areas, such as traditional medicines and diagnostics, would help to create trust between groups and build a stronger health innovation system.

## Background

The first African country to gain independence (from the British in 1957), Ghana has long been seen as a leader in Africa. With its plentiful natural resources (cocoa, gold, and recently-discovered oil), stable governance situation, long-standing universities and research institutions and improving communications infrastructure, many factors are in Ghana’s favour when it comes to economic development. Recent years have indeed seen improvements in Ghana’s macroeconomic environment - growth was roughly 6% in 2008 and 2009 [1]. In 2009 the World Bank rated Ghana as the best place to do business in West Africa [2], in part due to the stable political system which has increased the confidence of investors.

Science and technology (S&T) have formed part of Ghana’s development vision for decades, dating back to 1964 when the government under president Kwame Nkrumah published its ‘Seven year plan for national reconstruction and development’ which saw S&T as central to industrialization [3]. Much more recently, Ghana’s guiding policy documents cite the importance of S&T for improving productivity and socioeconomic outcomes [4-6]. Nevertheless, the concerted application of S&T to improve development has generally been elusive. Investment in universities and research institutions is low, and several studies still show Ghana to be a poor adopter, user and generator of technology [7,8]. Foreign firms cite Ghana’s lack of human resources, poor infrastructure, and low levels of technology as their main barriers to increased foreign investment [7].

One area where S&T has great potential to improve outcomes is health. Disease burden remains high in Ghana, with malaria the main cause of mortality and morbidity, accounting for about 21% of under-five deaths and 37% of all hospital admissions [9,10]. Other common diseases include cholera, tuberculosis and respiratory tract infections and there are currently around 260,000 people living with HIV/AIDS in Ghana [11]. Endemic neglected diseases include schistosomiasis, onchocerciasis (river blindness) and lymphatic filariasis (elephantiasis). Maternal mortality remains one of Ghana’s most pressing health problems and has recently been declared a ‘national emergency’ after indicators worsened [12].

Harnessing S&T to develop appropriate and affordable health solutions for Ghana, and indeed the wider West African region, has the potential to contribute to improved health outcomes as well as to offer opportunities for entrepreneurial activity that could add dynamism to the economy. Currently, Ghana’s health sector has a mix of both pharmaceutical and traditional herbal or plant medicines (traditional medicine encompasses a range of knowledge, beliefs and practices [13,14] but here we use it to refer to herbal or plant remedies for specific diseases). Drugs are predominantly imported and are estimated to constitute 60 - 80 % of the cost of health care in Ghana [15]. Plant medicines, which are typically cheaper than pharmaceuticals, are also easier to access in rural areas of the country [16].

In this paper, we present research on science-based health innovation in Ghana, including biotechnology. By science-based health innovation, we mean technological innovation across a spectrum of sophistication, from vaccines, pharmaceuticals, and medical devices to some plant medicines where attempts to scientifically standardize or characterize medicines have been made. We take a broad definition of innovation as not only new-to-the-world innovation, but also the diffusion, adaptation and use of technologies. We use the OECD definition of biotechnology: ‘the application of science and technology to living organisms, as well as the parts, products and models thereof, to alter living or non-living materials for the production of knowledge, goods and services’ [17].

The purpose of this paper is to describe and analyze science-based health innovation using an innovation system framework, which takes into account the wide variety of stakeholders who contribute to the innovation process and emphasizes the dynamic interaction and knowledge flow between them [18].  To date there has been little research on science-based health product innovation in Ghana, the exception being Essegbey’s 2003 innovation systems study on Ghana’s biopharmaceutical capacity [16]. This work builds on that study and was undertaken at the invitation of the late Major Courage Quashigah, former Minister of Health, Ghana.

## Methods

A case study research methodology was used in this study [19] . Data were collected through reviews of academic literature and policy documents and through open-ended, face-to-face interviews in Ghana. Interviewees were identified through purposive and snowball sampling; we interviewed 48 individuals from across the science-based health innovation system, including government officials (n=7), researchers (n=18), entrepreneurs (n=10), international donors (n=9) and non-governmental organization representatives (n=4) over three visits to Ghana from February 2007 to August 2008. We held a workshop in Ghana to discuss our initial findings in August 2007 and continued to engage stakeholders to address some of the challenges identified by this study throughout 2008 and into 2009.

When data for this study was collected, the government was that of the New Patriotic Party (NPP) under President Kufour. On 7 January 2009 President Atta-Mills and his New Democratic Congress (NDC) party assumed power. The results presented here are from the NPP period but, where significant developments have since occurred under the NDC, these are indicated.

All quotes are from the interviews unless noted, and with permission. This study was approved by the Office of Research Ethics of the University of Toronto.

## Results and discussion

In this section we discuss the roles of some of the principal actors in Ghana’s science-based health innovation system as identified by preliminary research and by the general innovation systems literature - government, research institutes and universities, the private sector, and non-governmental organizations (NGOs) and donors.

### Government

Government policies have been shown to play a large role in establishing an environment conducive to innovation in other developing countries, helping to set S&T priorities and funding levels, regulate technologies and support private sector growth [20,21].

Ghana has a variety of policies that link science to development and growth objectives, and that could therefore have implications for the application of health research to solve local problems. For example, Ghana’s first National S&T policy was developed in 2000 and outlined measures to address development challenges in areas such as health, agriculture and ICT using ‘innovative and modern technologies’, including biotechnology. It also aimed to improve the S&T environment through, for example, better technology education and increasing awareness of intellectual property rights [22]. Ghana’s Poverty Reduction Strategy II (GPRS II), which sets the target for Ghana to achieve a per capita income of at least US$1000 by the year 2015, sees S&T as underpinning industrial growth, encouraging the ‘adoption of appropriate technologies, both local and foreign, with the capacity to improve productivity and efficiency in the agricultural, industrial and services sectors’. However, our research showed little recognition of these aims in the health sector and weak implementation of S&T-driven goals. A number of interviewees in this study, including researchers at universities, were unaware that the 2000 S&T policy even existed and used its absence to explain poor levels of innovation in the health sector.

Government spending on research and development (R&D), including health research, has generally been fluctuating and low.  Though statistics are difficult to obtain, it appears that funding levels were higher in the 1970s and 1980s than the next two decades [23]. Adarkwa cites spending between 0.3% and 0.35% of GDP for 2002-03 [24], and UNCTAD in 2003 noted that “[a]llocations to the Ministry of Environment, Science and Technology constitute only about 0.25 percent of the GDP, a reflection of the importance accorded to science and technology and the environment. Moreover, only about two-fifths of the Ministry’s budget is used for research” [25]. This is far short of the African Union commitment of 1.0% minimum allocation of national budgets, and instead most funding for health R&D is from foreign sources.

Our research suggests a lack of strong leadership around S&T and poor co-ordination between the various Ministries as explanations for these funding and policy implementation challenges. This is borne out by the changing status of science at the ministerial level. In 2000, the relevant Ministry was that of Environment, Science and Technology (MEST); during our study, S&T formed part of the Ministry of Science, Education and Sport; since the NDC government took power, MEST has been reinstated.

In the health sector, priorities and programmes are set by the Ministry of Health (MOH), which had a budget of~$500m USD in 2007 [26] (approximately 14% of the Government budget that year), most of which was on health workers salaries. Ghana’s current 5-year program of work (2007-2011) highlights a range of goals including addressing communicable diseases such as HIV and malaria, reducing risk factors associated with non-communicable diseases, scaling up high-impact health interventions in areas such as nutrition and maternal health. A major focus has been on improving access of health services to the poor. Up until recently, patients paid out of pocket according to a ‘cash-and-carry’ system for access to health services. However, a National Health Insurance Scheme was put in place in 2004 with the aim of improving financial access to quality health services [27].

The MOH funds health research, largely through the Health Research Unit of the Ghana Health Service, the executive arm of the MOH, which has three field stations across the country (Navrongo, Kintampo and Dodowa). These sites have been involved in some exceptional examples of public health research — for example, the Vitamin A supplementation trials carried out at Navrongo until 1992 were some of the largest field trials ever carried out in Africa, and showed a 23% reduction in child deaths. The findings led to changes in national policy as well as policies of international agencies such as the WHO, UNICEF, and World Bank [28], which demonstrates that links between government and research exist, as well as links into the global donor community. Our research suggests the MOH is less strongly connected to other sources of health knowledge, such as the universities, though links do exist. The main challenge for innovation is the weak relationship between MOH priorities and the local productive sector.

In an attempt to address this problem, the MOH’s Health Research Agenda put forward the idea of supporting a ‘health industry’ – microbusinesses that improve public health through the provision of a product, service or intervention [29]. This approach is viewed partially as a mechanism to supplement an overstretched health budget, but also reflects dissatisfaction that almost all health products used in Ghana are imported— most allopathic drugs, all ITNs (insecticide-treated nets) and even hospital beds. Eddie Addai, a former Director at the MOH, acknowledged that, in terms of public health interventions, ‘what we haven’t done as a country is approach [interventions] from a business perspective. We’ve approached them from a science perspective. And we’ve left the business to grow, as it were’.

A fund of ~US$300,000-400,000 was set aside for these activities in the year 2006-2007, aiming to support novel ideas for translation of health research. However, limitations to this initiative proved considerable - personnel and time constraints within government, a lack of business knowledge within the Ministry, and an absence of links to other Ministries with an industry focus, and of links to industry itself. The fact that this ‘out-of-the-box’ policy was developed and integrated into the Ministry’s plans for research is encouraging; however in practice resource constraints and the precedence of other health policies means than there is little room for policy experimentation and novel ideas remain isolated ones.

Again, lack of alignment between government Ministries, aims and policies can be seen though the example of the local production of health products. In terms of procurement, the government gives a 15% price preference to local producers and Ghana’s National Drug Policy acknowledges that local manufacture has been weighed down by free market policies and seeks to support the private sector through funds to develop the raw material base for the pharmaceutical and herbal industries; technical support; and the encouragement of exportation. On the other hand, GPRS II does not highlight pharmaceuticals as a key sector for support, though it does aim to improve availability and access on a sustainable basis.

Alongside the allopathic or biomedical system, traditional medicine remains highly popular in Ghana. At least 60% of Ghanaians use traditional medicine as the first port-of-call in primary care, particularly in rural areas: ‘every village or hamlet has a traditional resource for health care within easy access’ [16]. The MOH therefore considers improving traditional medicine quality and standardizing practice as a potential route to considerable health returns, and has put in place a number of policies to encourage this over the years. This has been consolidated in a comprehensive National Strategic Plan for Traditional and Alternative Medicine Development 2005-2009, based on WHO resolutions and conventions. In practice this Plan sets an ambitious agenda, encompassing a range of stakeholders from traditional healers to policy-makers, and setting a large number of diffuse targets – all of which makes the Plan challenging to implement. Again, there are examples of disjointed policies: there are no TMs on the Essential Drugs List, for example, despite the fact that the Government seeks to promote their use.

As in all countries, regulation in Ghana is an important aspect of health innovation, setting standards for product quality and safety, and influencing access to markets. Health products in Ghana – including biomedical drugs, traditional medicines, and medical devices (e.g. bednets, diagnostics, condoms) whether locally manufactured or imported – are regulated by the Food and Drugs Board (FDB). The FDB also regulates the conduct of clinical trials. For traditional medicines, the FDB seeks evidence of safety over a two-year period but efficacy testing is not required, making regulation of plant medicine fundamentally different from pharmaceutical development. Though counterfeit drugs are a problem in Ghana, they are considered to be less so than in other countries in the region [30].

Our research suggests that the Ghanaian FDB is amongst the strongest in West Africa, with well-trained and active staff. A number of firms in our sample singled out the FDB as a helpful government unit. Drug testing is carried out at the National Quality Control Laboratory (NQCL) under the FDB. The FDB has a 5-year plan to upgrade infrastructure, including a new National Quality Control Lab which they will seek to accredit with the World Health Organization (WHO) and International Organization for Standardization (ISO). The FDB also offers Good Manufacturing Practice (GMP) training for firms, a process they are trying to encourage so that firms can better meet international standards. There is also a collaboration with USAID (with technical assistance provided by Unites States Pharmacopeia), aiming at building capacity to monitor the market for illicit and sub-standard drug. However, funds are restricted, and progress in these areas sporadic [31]. Regulators also face a challenge in balancing a push towards international GMP standards with supporting local firms that will need time and assistance to meet such standards.

The FDB is strongly involved with other countries in the region: it has helped to train regulators in Sierra Leone, for example, and works with the Economic Community of West African States (ECOWAS) through which a regional regulatory body for West Africa has been discussed. Our study showed strong support for regulatory harmonization; among other benefits, access to a market of 270 million people would be a major incentive for local health innovation. Ghana and Nigeria have similar drug regulatory structures based on the US FDA system, and we observed that personal linkages between executives of the FDBs in both countries meant that discussions on regulatory harmonization have taken place.

Another important policy area for health innovation is intellectual property regulation. Ghana has been a Member and Signatory to the TRIPS (Trade Related Aspects of Intellectual Property) Agreement since January 1995. Ghana is not considered a Least Developed Country by the World Trade Organization, and as such cannot take advantage of time extensions for TRIPS compliance. The 2003 Patent Act therefore amended Ghana’s previous Patent Law of 1992 in order to ensure all TRIPS obligations were met. An IP office exists in the Registrar-General’s office; however, our evidence showed very low levels of patenting of indigenous research, including in health. The knowledge, systems and costs needed for patenting mean it is simply not a realistic or relevant proposal for researchers. One interviewee said ‘the process is too cumbersome. For developing countries, I think it will take some time to get people patenting; it’s going to take a lot of time and energy’. This applies also to the field of traditional medicine where the question of how to protect indigenous or community knowledge is very complex. As yet there is no specific protection for indigenous knowledge in Ghana, though draft regulations were prepared in 2007.

Instead, interviewees associated IP in Ghana with global regulation which can restrict access to medicines. Ghana’s 2003 Patent Act includes some provisions which may improve access to medicines (such as parallel importation of lower-cost pharmaceuticals) and some which may not (doubling the time for patent protection to 20 years) [32].  However, sufficient legal grounds exist to use compulsory licensing to address its public health concerns, a situation that has been demonstrated by one Ghanaian firm which is making 7 Anti-retrovirals (ARVs) under compulsory licenses.

### Research institutes and universities

Our research showed that there are several Ghanaian institutions involved in international health research, largely in partnership with overseas universities and donor organizations. However, there is evidence that the capacity to train students to a high level, important for technologically-intensive areas, remains small. In 2002 Ghana’s higher education system contained just 127 PhD students [33] and S&T numbers in the system have decreased [34]. A lack of qualified applicants, limited laboratory infrastructure and poor job prospects are contributing factors in this decline [35].

#### University of Ghana

The University of Ghana gained full university status in 1961, and now has nearly 30,000 students (including roughly 1,800 graduate students) [36]. It has a number of departments and institutes relevant to biotechnology and to health, foremost among them the Noguchi Memorial Institute for Medical Research, which has roughly 260 staff working on communicable disease, nutrition, and non-communicable diseases (e.g. cancer, sickle cell, diabetes). Noguchi hosts a variety of laboratories for neglected diseases including a WHO-accredited polio laboratory, the lymphatic filariasis support centre for Africa, a clinical trial facility and rotavirus reference laboratory. Researchers are involved in a range of local and international collaborations exist – with the Ministry of Health, Ghana AIDS Commission, Yale University Medical School, Duke University, and Tokyo Medical and Dental University, to name a few.

According to the University’s annual report for the academic year 2007/2008, researchers across the University published about 50 health research papers in international journals and 7 in African ones; the highest number of publications was on HIV/AIDS, Buruli Ulcer and malaria [37]. Interestingly, almost without exception health research projects at the University, including those at Noguchi, were funded by external agencies including World Health Organization, the Department for International Development (UK), USAID, UNICEF, and the Bill & Melinda Gates Foundation.

Despite the volume of health research, its application and commercialization is limited. Our research showed an absence of patenting, industrial links and technology transfer capacity.  A pertinent example of this is the case of a dipstick test for urinary schistosomiasis (the second most prevalent parasitic disease after malaria), which was developed by researchers at Noguchi. Based on monoclonal antibody technology, the test was developed to prototype stage but no further. One of the researchers involved cited lack of a reward system for innovation, uncertainty about profitability, and the reliance on imported solutions to local problems as specific barriers to product development. It could be added that lack of demand from the private sector – either locally or elsewhere – is another reason for the stagnation of potential health solutions within laboratories.

#### Kwame Nkrumah University of Science and Technology (KNUST)

KNUST trains students and carries out research in a range of biological and physical sciences.  One of its main centers for health research is the Kumasi Centre for Collaborative Research, a joint venture between the Ministry of Health, KNUST and the Bernhard-Nocht-Institute for Tropical Medicine in Hamburg, Germany. The Centre acts as an international platform for biomedical research into tropical diseases, and contains modern laboratory facilities for parasitology, molecular biology and bacteriology.

As partners in the Malaria Vaccine Initiative, a global program of the international nonprofit organization PATH to accelerate the development of malaria vaccines, the Centre and the KNUST medical school are testing the first promising malaria vaccine product from GlaxoSmithKline, RTS,S (together with 8 other sites in Africa). Phase II trials showed that RTS,S was efficacious for at least 18 months in reducing clinical malaria by 35 percent, and severe malaria by 49 percent [38,39]. Phase III began in May 2009 and is scheduled to take 2 years and enrol up to 16,000 patients. The project is an example of how involvement with practical global health initiatives can enable capacity building: the Centre has built clinical trial capacity in the Kumasi region, enabling it to retain locally-qualified technical staff and offer them training opportunities in Good Clinical Practice and Good Laboratory Practice. These types of opportunity are vitally important for helping to stem the brain drain from health institutions. Professor Kruppa, the Centre’s Director, highlighted that in the first year of KNUST BSc course in lab technologies, ‘*t*he first batch after three years went completely abroad. Every single person went abroad*’*.  Other findings support this; it is estimated that 47% of Ghana’s college-educated graduates living abroad [40] and around 70% of Ghanaian medical officers trained in the 1990s have left the country [41].

In another example of locally-generated knowledge translation making a major impact on public health, the Centre, in conjunction with international partners, has developed a new treatment regimen for the diseases onchocerciasis (river blindness) and elephantiasis, which is caused by worms spread by the black fly. After clinical trials and publication [42,43] this approach has spread to other developing countries in Africa and Asia and received funding from the Bill & Melinda Gates Foundation, which aims to make the treatment regimen compatible with mass drug administration programs.

Technology transfer does occur at KNUST, largely at a micro-scale and around ‘low-tech’ products, with the help of its Technology Consultancy Centre, a long-standing group which fulfills ‘the university’s third role: that of service to the community‘[44]. Examples include projects in engineering, nutrition and sanitation, working strongly with the local community and to some extent with the Ministry of Health. Staff at the Centre see numerous opportunities for further knowledge translation and commercialization in health, though they have historically been hindered by a severe lack of resources and of links with the professional private sector. Our research found evidence of frustration that the government is failing to challenge local scientists to come up with solutions, and instead seeking technical expertise from outside the country.

#### Centre for Scientific Reseach into Plant Medicine (CSRPM)

Research in traditional herbal medicine is well-established in Ghana, with CSRPM, established in 1975, the main public research body. The Centre conducts research in phytochemistry and toxicity, both for regulatory and quality control purposes, and has facilities to carry out animal studies. Focus is on the safety of products, relying on in-house safety studies as well as documented and community ethnobotanical knowledge. Efficacy studies extend to in vivo animal testing but there have been no full-scale human clinical trials, which are hugely costly and not required by the FDB, which approves herbal medicines by relying on safety, not efficacy, data.

CSRPM has developed about 35 products, treating indications such as asthma, sickle cell anemia, and malaria, which it distributes through its clinic, which at busy times sees up to 200 people per day. One product, for the treatment of arthritis, is exported to Europe and the US.

No intellectual property has yet been generated and Dr Okine, CSRPM’s Director, recognizes that ‘there are grey areas of IP rights, especially with natural products’. At the point of interview, the Centre was attempting to work out IP modalities for the knowledge it receives from herbalists, acknowledging that traditional medicine is for the most part community knowledge. There is no national guidance on this issue yet.

Unlike research groups in the biomedical sphere, the CSRPM has managed to spin out its health knowledge to commercial ends. It also seems to be well-linked to a number of other groups in an exchange of knowledge. It has links to local traditional healers as well as to firms in the beverage industry, for which it produces plant extracts for flavoring, such as that from African mahogany (*Khaya senegelensis*). The on-site clinic also suggests stronger links to end users than in the pharmaceutical sphere. Finally, the Centre accepts interns from KNUST’s herbal medicine course and links them to local herbalists.

#### Council for Scientific and Industrial Research

The Council for Scientific and Industrial Research (CSIR) was founded shortly after independence, and later re-established by CSIR Act 1996 (Act 521) with a mission ‘to generate and apply innovative technologies which efficiently and effectively exploit Science and Technology for socio-economic development in the critical areas of agriculture, industry, health and environment, and improve scientific culture of the civil society’. CSIR comprises 14 research institutions, employs ~3,800 staff and is mandated to coordinate scientific research across the country as a whole. Eight of CSIR’s institutes cover agriculture; the others address animal, water, industrial research, construction, S&T information and science policy research. Although none of CSIR’s Institutes focus specifically on health, the potential outputs of agricultural and water research, for example, could have large indirect health impacts through improved nutrition and sanitation.

An interesting aspect of CSIR’s work with lessons for the role of innovation in Ghana is its efforts to align with the needs of industry, and to generate 30% of funding from external sources such as commercial activity and consultancy (a funding stream known as Internally-Generated Funds or IGF). Currently a commercial director exists to stimulate technology transfer and make CSIR’s research more appealing to the private sector. However it was conceded by interviewees that this was a very difficult goal to achieve – lack of strong links with industry and understanding by researchers of the mechanisms of product development have hindered the process, and the policy effectively puts the burden for ‘commercialization’ onto individual researchers, without structural support.  Furthermore, services were the main route to income generation, not necessarily adding to the knowledge base and reducing time available for research.

### The private sector

#### Pharmaceuticals

Pharmaceutical production in Ghana is 50 years old, as old as the country itself. There are approximately 40 pharmaceutical companies in Ghana as of 2009 and the number is growing. These include both foreign firms and locally-owned producers such as Starwin, Kinapharma, Ayrton Pharmaceuticals and Ernest Chemists. Local firms are almost entirely focused on generic over the counter medicines such as antibiotics, analgesics, and anti-infectives. Branded products (i.e. generic products branded by a company) are also popular and present a way for firms to distinguish their products from many similar ones. It is believed that due to an oversupply of production and fierce competition most firms are producing under capacity [15]. The pharmaceutical market is approximately 30% locally produced and 70% imported products.

A few firms are producing essential medicines, though these are largely provided by donors funds and sourced internationally. For example, anti-retrovirals (ARVs) are largely supplied by Indian companies such as Cipla and Ranbaxy and anti-malarials supplied by firms in India and China. It is difficult to obtain statistics for R&D in the private sector, but our evidence suggests that little formal R&D is taking place, mainly in the area of internal quality control.

DanAdams Pharmaceuticals, founded in 2004, is currently the only ARV manufacturer in Ghana – indeed, in the Economic Community of West African States (ECOWAS) region as a whole. Started as a joint Chinese-Ghanaian venture, as of 2007 it became a wholly Ghanaian enterprise focused on producing medicines again HIV/AIDS, malaria and other infectious diseases. Its founder, Yaw Gyamfi, a US-trained pharmacist, returned to Ghana motivated to address drug shortages in the country. Revenues generated from a community pharmacy practice, still in operation, were used to build a manufacturing facility, and the firm now manufactures 13 generic ARVs as well as antimalarials, anti-TB medications, analgesics and other drugs used for treatment of opportunistic infections in HIV/AIDS patients, and functions at GMP standards. Most of the initial ARVs the firm began by manufacturing were off-patent, but later ones, such as second-generation ARVs to be given to patients who were building resistance, are manufactured under compulsory licenses sought by the Ghanaian government. More recently, DanAdams has negotiated voluntary licenses – for example, it has an immunity-from-suit agreement with Bristol Myers Squibb for certain products.

The company has developed a number of innovative drug formulations, such as Camosunate PED, a paediatric antimalarial (containing amodiaquine, artesunate and paracetamol) produced in sachet form for ease of use. Camosunate PED is sold in Cote d’Ivoire, a rare example of penetration of the Francophone market, and is registered in a range of other West African countries. DanAdams has authorisation to market its ARVs in Burkino Faso, Sierra Leone, Liberia, Cote d’Ivoire and Ghana. However, WHO pre-qualification has become the main focus of efforts as it is a prerequisite of donors, such as the Global Fund, seeking to procure essential medicines.

LaGray Pharmaceuticals, founded by returnees from the Ghanaian and Nigerian diasporas, is also pushing boundaries. It has set up an international-standard manufacturing facility for the production of active pharmaceutical ingredients (APIs) – the only such facility in sub-Saharan Africa outside of South Africa – in order to increase self-sufficiency of drug production for the region, and to counter the fact that there are a large number of poor-quality imports. Currently, all APIs used in Ghana are imported, mainly from India. LaGray’s current product portfolio includes azithromycin, dermatologicals for opportunistic bacterial and fungal infections, and anti-infectives, with anti-retrovirals, anti-malarials and antihypertensives in the pipeline. In time, the firm is interested in manufacturing drugs for chronic disease indications such as diabetes. Prices are marginally less than imports, and LaGray uses specially-made hologram boxes to reduce possibility of counterfeiting of their products. LaGray is seeking to access regional markets, and have products filed with regulatory authorities in Nigeria, Togo, Benin, Senegal, and Cote D’Ivoire.

#### Plant medicines

The majority of all interviewees across the whole innovation system felt that the most promising area of health innovation for Ghana was in herbal or plant medicine, and that the current state of innovation in the field did not do justice to Ghana’s knowledge and potential in this area. Partly this is due to lack of linkages between researchers and industry. Yaw Gyamfi, Dan Adams’ CEO, acknowledged that firms and researchers did not interact. He pointed out that, for the future, ‘if there’s any collaboration at all, it has got to be in the area of research and development, whereby we’ll be looking at not only orthodox medication but herbal, because that is the future of our country*’*.

There are other challenges too. Phyto-Riker, formerly GIHOC Pharmaceuticals (a government-owned pharmaceutical company set up in 1968 to supply local and regional markets) has considerable manufacturing capacity in solid and liquid dose forms and is GMP and ISO 9001-2000 certified. At one point it was strongly interested in plant medicine and produced an anti-malarial teabag based on the herb *moringa*, which was to be further developed through R&D. However, financial and management constraints meant that this idea was abandoned, and the current CEO questioned whether it was ‘worth the risk’ to move into innovative products.

A more typical and very interesting approach to ‘scientific upgrading’ can be seen in the example of the Plant Medicine Company Ltd. Incorporated in 2000, it was founded and funded by a professor of organic chemistry interested in making standardized traditional medicine products of higher quality than are widely available, though without efficacy trials. For the Plant Medicine Company, the aim is to produce products such as those for pain relief, diabetes and immune boosting – mostly for local and regional markets, but also international ones. Unlike pharmaceutical-based companies, this firm has a direct link into the farming of plant products. Indeed, the main barrier identified by the CEO to sustainable commercialization was acquiring plant materials. Taking the example of *moringa*, he stated that ‘a lot of people are planting it, but they don’t know how to harvest it well. You know there’s a way of harvesting it, a way of drying it, a way of storing it, and so on and so forth. So again that is our biggest problem for me in herbal medicine in Ghana’. Strong links into the market were obvious with the company and the CEO asserted that his familiarity with the local community would naturally help to bring him sales.

A recent pertinent development in terms of plant medicine product development is that an American-Ghanaian firm, Phytica, has secured $1m funding from the NDC government to conduct clinical trials for its first product: an anti-malarial derived from the roots of *Cryptolepis sanguinolenta*, a climbing shrub indigenous to West Africa [45]. It is intended that trials will take place through a public-private partnership with the Noguchi Institute. According to the firm, complete studies of toxicity, bioavailability and pharmacokinetics of the new formulation will be followed by a formal clinical trial with hundreds of malaria patients. Though at an early stage, this would mark a new approach to scientifically-upgrading plant medicine innovation, especially as it is funded by local government rather than foreign sources.

Across the different activities, we found very few firm-firm linkages in the health sector in Ghana. A common reason cited for this was lack of trust between groups, leading to an unwillingness to collaborate or share information. One CEO summed up this situation as ‘there’s still proprietorship, father and mother kind of business’. Firms were much more likely to have links with overseas firms, both in the North and the South, from which inputs, knowledge and, in some cases, financing is obtained. Nor could we find links between firms and the local research community, except that firms hire graduates and give them on-the-job skills training.

The environment for these science-based firms working in the health field in Ghana is difficult. Regarding financing, bank rates are considered far too high for securing loans at around 20-25% - one interviewee stated that ‘Ghana banks are not a place to look for money. They don’t understand. To them, it’s just a commercial venture, they don’t understand long-term profits’. Another described the lending rate as ‘highway robbery’. The firms we spoke to were largely self-funded through personal investments, or from other revenue sources such as services. If there was external funding, it was not from within Ghana. For example, it was the LaGray management’s status as dual nationals with the US that enabled them to access their initial investment from the Overseas Private Investment Corporation (OPIC).

While the Ghana Stock Exchange was considered reputable, it is not yet interested in technological ventures in the health field; rather, it focuses on lower-risk activities such as mining and real estate. Two local pharmaceutical companies have floated on the GSE (Ayrton and Starwin) but ‘the extent of capital attracted…is not substantial’ [30] as the sector is seen to be undermined by a reliance on international exports.

Venture capital is limited, and not attuned to the needs of a small business in a developing country. Rather, local venture capitalists reportedly adopt a ‘US model’, and expect fully formed companies before investing. The Government of Ghana Venture Capital Trust Fund (VCTF) has been set up to provide investment capital to SMEs, with pharmaceuticals as one of the priority sectors. However, to date the VCTF has not invested in the health sector. While for some firms lack of financing is a major barrier, others would rather grow slowly without the threat of interference. One interviewee, averse to venture capital, said ‘these people come in and try to dominate you. I don’t like that. I’d rather go at my pace and increase slowly, rather than saddling myself with debt, and [having] people to come and tell me what to do.’

The competitive advantage for most firms, when compared with those exporting products from India and China, was their location and knowledge of the local market. Firms had products registered all over West Africa, and a common theme expressed by interviewees was the competitive local market and the need for effective branding and marketing through television and radio. There was also intense interest in any mechanisms that allowed for easier penetration of regional markets, such as regulatory harmonization. Access to Francophone markets, is also a growing area where Ghanaian manufacturers might be well-positioned. One interviewee said that ‘the Indians have not yet understood the Francophone market, which is good for me’.

However, the practical logistics of accessing markets remain a major difficulty – the complexity of drug distribution meant that firms had to rely on building their own networks, which is an expensive and time-consuming endeavor. An NGO representative working on drug distribution reiterated this difficulty: ‘each and every one is developing his own distribution system, making it very expensive for products to get to the people who need them…my wish is that the market be consolidated and move away from the highly fragmented nature that it is now’.

One method to overcome this is to gain WHO prequalification and be eligible to sell products to international donors; a number of firms, already GMP qualified, were attempting to do this. However, it is an expensive (~$500,000) and cumbersome process. The need to prove bioequivalence (the measure of the equivalence of difference formulations of a drug in terms of bioavailability in the body) was another requirement causing difficulty for firms due to lack of any facility for this on the continent outside South Africa. A final point is that in general firms were not seeking patents; other forms of secrecy are more prevalent, such as trade secrets. Even if a patent was obtained, it would not necessarily be useful - costs would prohibit protecting any infringement, for example.

Despite the fact that Ghana has tried hard to make business attractive, few firms cited any support from government, except for a policy of a 15% preferential price adjustment to local firms and possible assistance with gaining ISO qualification. As the role of SMEs in the economy has risen in importance, so has the range of programs to support them financially and through training – e.g. Empretec, a non-governmental organization for support of entrepreneurs; the National Board of Small Scale Industries; and GRATIS, the public agency responsible for tech development and transfer. However, none of these were mentioned by our interviewees in the health sector; this may be because such initiatives tend to focus on ‘lower-tech’ health interventions, and the area of science-based business is poorly understood.

Firms identified a range of areas where potential government support could be improved, including VAT exemptions on a wider range of imported materials necessary for pharmaceutical manufacturing, and incentives to train staff and build capacity. One interviewee simply wished for less bureaucracy, saying ‘there are very cumbersome laws in Ghana, very basic things which have nothing to do with pharma production….they have to do with business.’ One example given was the Ghana Investment Promotion Centre (GIPC) law which was supposed to make the GIPC a ‘one-stop-shop’ for businesses, but this efficiency has been undermined because an earlier investment law had not been repealed. This highlights the difference between policy and practice often evident in Ghana: ‘if the GIPC law actually worked the way it’s supposed to work, it would be wonderful’.

In the face of these challenges, the entrepreneurial drive of the founders and their willingness to take risks with their own assets is clear. One Ghanaian CEO commented ‘people find me unusual, but I’ve always been entrepreneurial, from childhood’. Almost all the founders were African but had been educated or worked in the US or Europe, for varying lengths of time, attesting to some element of brain circulation rather than simple brain drain. These interviewees were driven towards entrepreneurship by a combination of factors: the market opportunity, a sense of challenge, and the strong desire to ‘give back’ to Africa’. One CEO said that ‘as a major shareholder, of course I want to maximize my investment, but at the same time I also want to open doors’.

### NGOs and donors

As one of the favored African countries for donor aid, the health sector in Ghana, including health research sponsored by the Ministry of Health, is heavily supported by outside funding. Of the 19 donors active in Ghana’s health sector between 2003 and 2005, the largest were the World Bank ($136.9 million), the Netherlands ($103.9 million), the United Kingdom ($71.4 million), the United States ($59.9 million) and Denmark ($37.8 million) [46]. Other philanthropic agencies are also directly involved in supporting the Ministry of Health’s research institutes. The Navrongo Health Research Centre, for example, is supported by Rockefeller Foundation, United States Agency for International Development (USAID) and National Institutes of Health (NIH), World Health Organization, Meningitis Research Foundation, the Canadian International Development Research Centre, and the Bill & Melinda Gates Foundation.

In the universities and research institutions, we found that almost all of the biomedical research conducted in Ghana, including biotechnology, was funded by national development agencies and multilateral donors.  Outside support for such research has a number of positive impacts in terms of knowledge generation, transfer of expertise, local training and capacity building. However, it can also be problematic. For example, donor priorities don’t necessarily align with those of the research community, and funding streams can be erratic and discontinuous; examples were found of projects in tuberculosis and in vector control which were abandoned after funder priorities changed.  Some lack of clarity was shown by interviewees around ownership of donor-funded research results, and, in the area of plant medicine, unease was expressed by a few interviewees who maintained that partners were further researching Ghanian plants once they had taken them out of the country. It is worth noting that a number of interviewees felt strongly that aid has made people dependent on outside solutions at the expense of local expertise.

Another interesting point with respect to health innovation is that donor support tends to end at the research level, and has not generally extended to assessing the commercial potential of research or aiding in technology transfer. Furthermore, while research institutions have benefited from financing, firms find it very difficult to access funds. This may be partly due to donor funding being frequently tied to ‘pro-poor’ efforts which will have direct effects on communities, rather than science-based initiatives which may make a longer-term contribution to economic development.

In terms of NGOs operating in the health product innovation field – as opposed to health services – we found very few. Technoserve, an NGO which supports entrepreneurial activities mainly in the agriculture sector in Ghana, had given a grant to one of the firms we interviewed to allow it to assess the value of laboratory services it was considering operating. Though largely outside the scope of this study, a number of NGOs are indirectly contributing to the health innovation system through providing vital access to rural markets, capacity-building and skills development. Two examples of note were MircoBusiness for Health, an NGO developing an innovative service model based around training women in rural villages to provide affordable preventative health products in rural communities, and INDEPTH, a not-for-profit which consists of 35 health and demographic surveillance system sites in 18 countries in Africa, Asia and Oceania. INDEPTH is known for its Young Scientist Capacity Building Program, which in collaboration with Witwatersrand University of South Africa offers a Masters degree in Field Epidemiology to young scientists of the network, and is being supported by The Bill & Melinda Gates Foundation to develop capacity the conduct of GCP-compliant malaria drug and vaccine trials. It has with this grant built or strengthened 11 clinical trial sites to be involved in phase III of RTS,S malaria vaccine trials in Africa [47].

Finally, the donor community is a very important market for health products, and their priorities, policies and procurement choices therefore affect the focus of local innovators. Drugs for diseases classed as ‘priority endemic diseases’ are almost entirely supplied by the donor sector, particularly the Global Fund to Fight Aids, Tuberculosis and Malaria [30], and largely sourced from Indian and Chinese firms.

## Conclusions

### Strengths and good practices

Ghana is active in several areas which could underpin science-based health innovation in the future. These include several health and biosciences research and training institutions with strong outside research linkages and donor support; a relatively strong regulatory system which is building capacity in other West African countries; the beginnings of new funding forms such as venture capital; and the return of a number of professionals from the diaspora, particularly in the private sector, bringing expertise and contacts. The attempt by the Ministry of Health’s interest to spur a ‘health industry’ also signals an openness to creative policy thinking which sees health not only as a social expense but also a productive sphere. Importantly, a variety of health products and services are already being developed in Ghana, which are innovative in the sense of being new to the country and, in some cases, the continent. They include essential medicines, raw pharmaceutical materials and new formulations for pediatric use.

Ghana’s activities in commercializing traditional medicine are perhaps especially worthy of note, as they encompass a wide variety of initiatives at different stages of development. Almost every interviewee in this study highlighted the perceived potential in this area, based on factors such as considerable local knowledge, increased government interest in the area, and the large market potential both at home and abroad.  Ghana is ahead of many other African countries in developing policies to improve standards in traditional medicine practice and products.

There is, however, a distinction between commercialization for local use, as is already occurring through institutions such as the Centre for Scientific Research into Plant Medicine, and producing products according to international biomedical standards, which has been a more elusive goal.Our study has focused on science-based innovation, and shows that a number of institutions exist that could contribute to scientific upgrading of traditional medicine Ghana’s capacity in this area, a finding borne out elsewhere [48]. The recent funding of a public-private partnership between a local firm and the Noguchi Institute for Medical Research to conduct clinical trials on a herbal anti-malarial is an interesting development that would represent a very different approach to traditional medicine product development However, the complexity of issues around ownership and benefit sharing still remain and will require considerable attention, not least the passing of the draft Bill for the protection of indigenous knowledge into law.

### Recommendations

Innovative health activities in Ghana are generally being driven by individual entrepreneurs relying on international linkages for access to physical inputs, knowledge and/or markets, who are not well-connected to other local firms, research organizations or government bodies (though there are exceptions such as the Food and Drugs Board). They have received little local financial or logistical support, and face major challenges to growth and even sustainability. A key concern for them and for other firms hoping to grow in this area is access to knowledge (local or international) and to markets, be they local, regional, or part of the global procurement system; each of these pose their own difficulties.

Health and biomedical research institutions are contributing to health innovation in a broad sense, primarily through providing training. However Ghana’s continuously low national support for health research has resulted in deteriorating scientific infrastructure and low morale of research staff working in the public sector, particularly in research institutions. Demand for research by industry has been poorly understood, public-private links are weak and public sector commercialization policies, which have focused on the supply side, have therefore been ineffective. The strong presence of donors in funding health research, while helpful in keeping science alive and supporting some excellent examples of knowledge transfer with public health implications, is liable to be short-term and has not been helpful in generating locally-owned knowledge or its local commercialization.

In Ghana, the challenge at the policy level is not so much the creation of policy documents, but rather in their implementation and co-ordination with other policy aims and objectives. A series of S&T policies with weak implementation has left researchers unmotivated, and different elements of the innovation system working independently without a coherent goal. Our research showed different attitudes and commitment to S&T across different government ministries, with no overarching leadership. A positive step is the recent reestablishment of a Ministry for Environment, Science and Technology, and the development of a national STI policy which explicitly considers innovation as well as S&T [49]. The challenge now is to link this to concrete outcomes and to economic and health goals.

While Ghana has a large number of institutions in the public and private sector concerned with health research and its commercialization, their ability to work together to address clear health goals is low. Some strong linkages are apparent – between health researchers and the international research community, between local firms and their (mostly international) suppliers, between NGOs and the community. However, the systemic aspect is absent and groups remain isolated from collectively mobilizing research towards economically-productive and sustainable ends. Lack of trust between stakeholders, unwillingness to share information, and different working cultures were issues repeatedly expressed by interviewees – issues which will only be overcome through concerted efforts to forge partnerships around health goals, and create incentives for firms to interact and share risk.

After our initial fieldwork in Ghana, we continued to work with stakeholders who constitute the health and biotechnology innovation sector. A working group on health and biotechnology innovation was established, with representation from government, the research community and the private sector. The group’s goals were to consider how to build a sustainable hub in Ghana that would help to link stakeholders, overcome barriers to collaboration, and stimulate locally-relevant health innovation. The group identified some key initial activities in this regard, such as setting up a virtual network to bring together stakeholders through the internet and regular meetings; a technology roadshow that would showcase Ghanaian technologies developed at research institutions to a wider audience, including financiers; and creating a database of technologies, infrastructure and equipment within Ghana that could be easily accessed and help to identify potential areas of cooperation. This novel approach could in the long-term lead to more demand-driven innovation in the health sector [50,51].

Based on our research, we suggest four recommendations to support health innovation in Ghana:

*Recommendation 1: Support small firms to build capabilities and access markets.* A broad range of incentives could be applicable here, such as support for firms to undertake R&D; better financing instruments such as lower banking rates and credit guarantee schemes; reducing taxes on raw materials; improving conditions for importing equipment; procurement of locally-developed products; and improved market access through better distribution systems and market knowledge. North-South and South-South partnerships for knowledge and technology transfer should also be encouraged. At a regional level, the possibility of regional regulatory harmonization for health products should be pursued. Finally, reviewing and removing old laws that are adding unnecessary bureaucracy to small businesses, such as the old Ghana Investment Promotion Law, would be beneficial.

*Recommendation 2: Increase national funding commitment to S&T, and consider what types of donor health research support could be most beneficial for innovation.*  Ghana should at least reach the 1% of GDP target for investment in S&T set by the African Union, and break the cycle of dwindling support for science. Updating key laboratory infrastructure where possible would be beneficial. A more strategic approach to commercialization is needed, perhaps around particular technology areas with incentives for joint public-private research and development. Dialogue with donors around the potential for health research to better be linked to economic and innovative activities and to generate locally-owned knowledge would also be beneficial.

*Recommendation 3: Set clear targets for implementation of STI policy for health and ensure coordination of policies across government Ministries* The most recent STI policy – which includes innovation for the first time – is a positive step. However, it remains broad and varied in its goals and does not set measurable targets for success around tangible health outcomes. If previous problems with implementation are to be avoided, specific activities around health and health technologies need to be identified and S&T, economic and health goals and policies need to be aligned. Thought should also be given to how to build a health industry which can employ, challenge, and mobilize qualified personnel – without local opportunities, brain drain will continue to negatively affect the country. For this, complementary policies will be needed around local manufacturing, procurement, health, access, and business support.

*Recommendation 4: Establish a focal point for health innovation.* Such a focal point would actively bring together relevant stakeholders from the government, research, private sector, donor and NGO communities. This could build on the activities of the working group on health innovation that was established during this study. Their work would need to go beyond a committee or policy decision, as the same issues around knowledge translation have been arising for many years in the Ghanaian context. As has been shown by other researchers of innovation in developing countries, ‘practical collaboration on concrete activities is essential’ [55], rather than overly ambitious projects or plans. In the health context, this could perhaps occur through a product development program around specific health products, such as diagnostics, bednets, plant medicines, or other nationally-determined health goals – perhaps linked to import substitution for commonly-used health products.

From our study, it can be concluded that Ghana’s entrepreneurs are setting the stage for health innovation activities, and that policies and support mechanisms need to respond to these activities to ensure they grow. The macroeconomic and political stability of the country, its strong health research networks and donor support mean that some fundamental factors are in Ghana’s favor in capitalizing on health innovation to address local needs, if they can be built upon. This is demonstrated by the growing numbers of diaspora returnees in the health sector who consider this the right time for Ghana to build capacity in essential medicine production. Crucially, mobilizing the large number of disparate groups involved in addressing local health is vital – not just as a social need, but also as an economic opportunity which can generate jobs and build self-sufficiency in health products. Recent activities, such as support for a public-private partnership around a plant anti-malarial, suggest that Ghana is ahead of the curve and building on its long experience of development with what have the potential to be groundbreaking initiatives.

## Competing interests

None declared.

## Authors' contributions

SAB, PAS, ASD contributed to the concept and design of this study and participated in site visits, analyzed the findings, and participated in manuscript development.
